# Childhood and Parental Asthma, Future Risk of Bipolar Disorder and Schizophrenia Spectrum Disorders: A Population-Based Cohort Study

**DOI:** 10.1093/schbul/sby023

**Published:** 2018-03-09

**Authors:** Qiong Wu, Christina Dalman, Håkan Karlsson, Glyn Lewis, David P J Osborn, Renee Gardner, Joseph F Hayes

**Affiliations:** 1 Division of Psychiatry, University College London, London, UK; 2 Department of Public Health Sciences, Karolinska Institutet, Stockholm, Sweden; 3 Department of Neuroscience, Karolinska Institutet, Stockholm, Sweden

**Keywords:** inflammation, etiology, epidemiology, risk factor, asthma, schizophrenia

## Abstract

**Background:**

Mounting evidence implicates early life and prenatal immune disturbances in the etiology of severe mental illnesses. Asthma is a common illness associated with chronic aberrant immune responses. We aimed to determine if asthma in childhood and parents is associated with bipolar and schizophrenia spectrum disorders.

**Methods:**

A cohort study including all children born in Sweden 1973–1995 (*N* > 2 million) assessing associations between childhood hospitalization for asthma, parental asthma during and pre-pregnancy, and subsequent bipolar and schizophrenia spectrum disorders.

**Results:**

Children with hospitalizations for asthma between 11 and 15 years had increased rates of bipolar (adjusted hazard ratio [aHR] = 1.73, 95% confidence interval [CI] = 1.21–2.47) and schizophrenia spectrum disorders (aHR = 1.62, 95% CI = 1.08–2.42). However, there was no association with asthma before aged 11. These results were supported by an analysis of siblings discordant for asthma. We found an association between both maternal and paternal asthma and bipolar disorder (aHR = 1.60, 95% CI = 1.27–2.02, and aHR = 1.44, 95% CI = 1.08–1.93, respectively), but not between parental asthma and schizophrenia spectrum disorders.

**Conclusions:**

As far as we are aware, this is the first study to find increased risk of bipolar disorder in children of individuals with asthma. Asthma admissions before aged 11 do not appear to be linked to bipolar or schizophrenia spectrum disorders. Taken together, our results do not suggest a straightforward link between asthma and severe mental illness via neurodevelopmental effects of inflammation, but potentially there is shared genetic vulnerability. This finding has implications for understanding the differential pathogenic mechanisms of bipolar and schizophrenia spectrum disorders.

## Introduction

Asthma is a common illness, with considerable medical and psychosocial morbidity and a substantial economic burden, characterized by episodes of breathlessness and wheeze.^1^ Symptoms are secondary to a chronic aberrant immune response within the airways.^2^ Among inflammatory atopic disorders, asthma is the most strongly and consistently associated with mental health problems in general.^3–5^ A small number of longitudinal cohort studies have found patients with asthma as adolescents have higher risks of developing bipolar disorder^6,7^ and schizophrenia.^4^ A study of the Danish population found that schizophrenia rates were also elevated in children with a first hospital contact for asthma in the under 5 age group and those aged 5–10 years after accounting for age, sex, calendar year, urbanicity, and psychotic family history.^4^ The existing studies of bipolar disorder are minimally adjusted for confounding factors, have limited follow-up beyond the peak age of bipolar disorder diagnosis and have not investigated the potential relationship between early childhood asthma and bipolar disorder.

Acute asthma during pregnancy is a serious complication associated with increased morbidity in offspring.^8^ Systemic increases in inflammatory markers such as IL-6 and C-reactive protein (CRP) have been reported in individuals with asthma^9,10^ and among pregnant women, serum CRP levels have been reported to predict offspring wheeze and asthma symptoms.^11^ Studies of maternal inflammatory states, with a range of underlying causes, suggest an association between immune response and mental illness, and there is evolving experimental evidence that the maternal and early childhood immune system abnormalities play a role in neurodevelopment.^12,13^ Investigated insults in humans associated with the development of psychotic illness include fetal exposure to maternal viruses and protozoa,^14,15^ autoimmune disorders,^16,17^ and neonatal abnormalities in acute phase proteins.^18,19^ The potential association between maternal asthma and mental illness has not been widely investigated. As far as we are aware, only one previous longitudinal study has investigated whether schizophrenia rates were increased in the offspring of mothers with asthma, and found a weak association, which was not sustained after adjustment for parental psychiatric history.^4^ The investigators found no association between schizophrenia and asthma among other first-degree relatives, suggesting little confounding by shared familial factors.^4^

We conducted a longitudinal cohort study starting during fetal life, to assess the temporal association between asthma and severe mental illness (SMI). We studied associations between childhood asthma and maternal asthma before and during pregnancy, and bipolar disorder and schizophrenia spectrum disorders. We also aimed to address the limitations of previous bipolar disorder studies by examining potential risk periods for asthma in childhood. We stratified children by age of first hospitalization for asthma, to determine if any associations are age-dependent. We hypothesized that asthma during pregnancy or early life would convey the greatest risk of bipolar and schizophrenia spectrum disorder, in keeping with neurodevelopmental theories.^20^ To explore the potential for confounding by shared familial factors, we additionally used a negative exposure control (fathers’ asthma history)^21^ and discordant sibling comparison approach.^22^

## Methods

### Study Population

Data were collected from national registers of Sweden from 1 January 1973 until 31 December 2011. For the purpose of this study the Total Population Register, Medical Birth Register, Multi-Generation Register, Migration Register, the Longitudinal Integration Database for Health Insurance and Labor market studies, Cause of Death Register and National Patient Registers were linked (inpatient records began in 1964 and outpatient in 2001, with full population coverage by 1973 and 2006, respectively). These registers contain sociodemographic and medical information on each resident of Sweden, and parents can be linked to children via a unique identifier. We included all children born in Sweden from 1973 to 1995, and their parents in the cohort. Ethical approval for the study was obtained via the Regional Ethics Committee at Karolinska Institutet, Stockholm.

### Asthma

Childhood asthma was grouped by age of first inpatient admission; ≤5years, 6–10 years, and 11–15 years of age. We identified children born to mothers who required inpatient admission for asthma before their birth, and during pregnancy and those who stated they had a previous diagnosis of asthma at their antenatal screening (recorded in the Medical Birth Register). Paternal asthma admissions before offspring birth were also identified. Diagnoses were made by the attending physician according to the Swedish version of the *International Classification of Diseases* (*ICD*) revisions 8 (1969–1986), 9 (1987–1996), or 10 (1997–present). Full ICD codes for asthma are given in supplementary table 1.

### Bipolar Disorder and Schizophrenia Spectrum Disorders

Diagnoses were based on ICD-8, ICD-9, and ICD-10 codes consistent with bipolar disorder and schizophrenia spectrum disroders (schizophrenia, or other nonaffective nonorganic psychoses) (full codes in supplementary table 1) recorded in inpatient or outpatient records. If an individual received more than one of these codes we used the last one in their record (when the treating physician would have had the most information about the longitudinal course of the disorder). We group all bipolar disorder diagnoses together and all schizophrenia spectrum disorders together. Individuals with a diagnosis of bipolar disorder or schizophrenia spectrum disorder, before age of 15 were excluded from analysis.

### Other Covariates

Potential confounders were identified a priori based on previous literature that suggested associations with both asthma and bipolar disorder or schizophrenia spectrum disorder risk. We included sex, birth order,^23^ socioeconomic status (SES) (defined as quintile of family income at birth, based on the whole population of Sweden),^24–26^ urban or rural residency,^27,28^ whether the mother was Swedish born, preterm delivery,^29,30^ antenatal infections,^14^ childhood respiratory infections before the age of 6 (using codes included in supplementary table 1), maternal and paternal age,^31–33^ maternal and paternal asthma admissions before the child was born (for mutually adjusted models),^34^ and parental history of SMI (including inpatient diagnoses of bipolar disorder, schizophrenia, schizoaffective disorder, and other nonaffective nonorganic psychoses).^35^ Age and calendar year were included as time-varying covariates. Maternal smoking data were available from the Medical Birth Register for children born from 1982 onwards.

### Statistical Analyses

Cox proportional hazard regression models were used to investigate the relative hazards of receiving a diagnosis bipolar disorder or schizophrenia spectrum disorder, during follow-up, from the age of 15. Participants were followed-up until the earliest of: first bipolar disorder or schizophrenia spectrum diagnosis, emigration, death, or end of follow-up (December 31, 2011). After running an unadjusted univariable Cox regression model for each exposure, we constructed a number of multivariable models. Firstly, we adjusted for sex, and time-varying age and calendar year (Model 1). For the childhood asthma exposure, we additionally adjusted for SES, urban born, mother Swedish born, premature birth, birth order, maternal hospitalization due to infection during pregnancy, hospitalization due to respiratory infection before aged 5, parental SMI, maternal age, paternal age, maternal asthma history, and paternal asthma history (Model 2). For the parental asthma exposure, our fully adjusted model accounted for age, sex, calendar year, SES, urban born, mother Swedish born, birth order, mother’s hospitalization due to infection during pregnancy, parental SMI, maternal age, paternal age, and other parent’s asthma status before birth (Model 3). In each case, we applied a cluster sandwich estimator to obtain a robust variance estimate that is adjusted for within-family correlation.^36^ The proportional hazards assumption was checked using Schoenfeld residuals.^37^

To check the validity of our results, we performed a number of pre-specified supplementary analyses. To account for the potential effect of bipolar or schizophrenia spectrum prodromal symptoms on asthma admission we excluded individuals developing these disorders before their 17th birthday. We examined the relationship between paternal asthma before the child was born and the risk of offspring bipolar disorder or schizophrenia spectrum disorder. We hypothesized that if there was a causal association between maternal asthma and offspring bipolar or schizophrenia spectrum disorder that acted through maternal and fetal immune system activation or other specific materno-fetal pathways, there would not be a similar association for paternal asthma and this would act as a negative exposure control.^38^ We also completed a discordant sibling analysis where we identified full siblings discordant for asthma before the age of 15 in each family, and assessed their rates of bipolar disorder and schizophrenia spectrum disorder. This method was developed as a way of controlling for shared genetic factors and familial environment.^22^ In this discordant siblings cohort, our fully adjusted stratified Cox model only included covariates that could vary between siblings. We repeated our fully adjusted models in a restricted cohort (1982 onwards) including maternal smoking status as an additional confounder. Data processing and analyses were performed using Stata 14.^39^

## Results

### Cohort Characteristics

The cohort consisted of 2258098 individuals born in Sweden between 1973 and 1995. Of these, 40187 (1.78%) children were admitted with asthma before their 15th birthday and 9892 (0.44%) children were born to mothers who were admitted with asthma admissions before their birth. During follow-up, 12705 (0.56%) individuals were diagnosed with bipolar disorder and 9940 (0.44%) individuals were diagnosed with schizophrenia spectrum disorders (3325 of these were schizophrenia diagnoses). Table 1 shows baseline characteristics of this cohort.

**Table 1.** Baseline Characteristics

	No Maternal Asthma	Maternal Asthma Prebirth	No Asthma ≤15 years	Asthma ≤5 years	Asthma 6–10 years	Asthma 11–15 years
Overall population, *N*	2248206 (99.56)	9892 (0.44)	2217911 (98.22)	32263 (1.43)	4929 (0.22)	2995 (0.13)
Male sex, *N* (%)	1154514 (51.35)	5101 (51.57)	1133613 (51.11)	21186 (65.67)	3122 (63.34)	1694 (56.56)
Mother Swedish born, *N* (%)	2005417 (89.20)	9299 (94.01)	1978769 (89.22)	28889 (89.54)	4396 (89.19)	2662 (88.88)
Maternal age at birth, median (IQR)	28.24 (24.93–31.88)	27.75 (24.26–31.76)	28.24 (24.93–31.87)	28.38 (25.07–32.15)	27.74 (24.30–31.59)	27.45 (24.13–31.38)
Paternal age at birth, median (IQR)	30.71 (27.29–34.79)	30.27 (26.69–34.68)	30.71 (27.28–34.78)	30.95 (27.39–35.57)	30.35 (26.87–34.51)	30.18 (26.60–34.51)
Quintile of family income at birth, *N* (%)						
1 [lowest]	432625 (19.24)	1909 (19.30)	426462 (19.23)	6506 (20.17)	954 (19.35)	612 (20.43)
2	447585 (19.91)	2142 (21.65)	441082 (19.89)	6950 (21.54)	1046 (21.22)	649 (21.67)
3	448211 (19.94)	2080 (21.03)	441941 (19.96)	6715 (20.81)	1026 (20.82)	609 (20.33)
4	448380 (19.94)	1941 (19.62)	442639 (19.96)	6088 (18.87)	1006 (20.41)	588 (19.63)
5 [highest]	441683 (19.65)	1645 (16.63)	436588 (19.68)	5396 (16.73)	844 (17.12)	500 (16.69)
Missing	29722 (1.32)	175 (1.77)	29199 (1.32)	608 (1.88)	53 (1.08)	37 (1.24)
Urban born, *N* (%)	332833 (14.80)	1510 (15.26)	328081 (14.79)	5059 (15.68)	746 (15.13)	457 (15.26)
Missing	202 (0.01)	0 (0.00)	195 (0.01)	7 (0.02)	0 (0.00)	0 (0.00)
Preterm delivery, *N* (%)	122301 (5.44)	724 (7.32)	118769 (5.35)	3700 (11.47)	339 (6.88)	217 (7.25)
Hospitalization for respiratory infection aged ≤5, *N* %	206603 (9.19)	1803 (18.23)	189996 (8.57)	16656 (51.63)	1173 (23.80)	581 (19.40)
Maternal infection during pregnancy	37823 (1.68)	497 (5.02)	237193 (1.68)	949 (2.94)	125 (2.54)	53 (1.77)
Parental SMI, *N* (%)	45599 (2.03)	310 (3.13)	44888 (2.02)	812 (2.52)	127 (2.58)	82 (2.74)
Maternal asthma before birth, *N* (%)	—	—	9113 (0.41)	669 (2.07)	67 (1.36)	43 (1.44)
Paternal asthma before birth, *N* (%)	7149 (0.32)	69 (0.70)	6748 (0.30)	415 (1.29)	36 (0.73)	19 (0.63)

*Note*: IQR, interquartile range; SMI, serious mental illness.

### Childhood Asthma

In the unadjusted analysis, children with an admission for asthma before their 6th birthday, and those with a fist admission between 11 and 15 years old, had increased rates of both bipolar disorder and nonaffective psychosis (table 2). Following adjustment for important confounders (Model 2), there was no longer an association between asthma ≤5 and bipolar disorder or psychosis. However, there remained an association between first asthma admission aged 11–15 and both bipolar disorder (HR = 1.73, 95% CI = 1.21–2.47) and schizophrenia spectrum disorder (HR = 1.62, 95% CI = 1.08–2.42). Excluding individuals who developed bipolar disorder or schizophrenia spectrum disorder before aged 17 did not alter our findings for the 11–15 asthma group (HR = 1.53, 95% CI = 1.05–2.23 and HR = 1.50, 95% CI = 1.03–2.30, respectively). We identified 27485 children with asthma before 15 years old who had at least one sibling without asthma. A similar association was suggested by this discordant sibling’s analysis: siblings with asthma aged 11–15 had increased rates of schizophrenia spectrum disorders (HR = 2.87, 95% CI = 1.05–7.79) and the point estimate for bipolar disorder was elevated, but with wider confidence intervals that included one (supplementary table 2). Limiting the cohort to those born after 1982, and additionally adjusting for smoking status at first antenatal visit, had minimal effect on the point estimates for the association between asthma and bipolar disorder, but confidence intervals included one. There was no evidence of an association between childhood asthma and schizophrenia spectrum disorder after additionally accounting for smoking (supplementary table 3).

**Table 2.** Bipolar Disorder and Schizophrenia Spectrum Disorders in Individuals Exposed to Childhood Asthma

	Bipolar Disorder				Schizophrenia Spectrum Disorder			
	Cases Diagnosed/ PYAR	Unadjusted HR (95% CI)	Model 1, HR (95% CI)	Model 2, HR (95% CI)	Cases Diagnosed/ PYAR	Unadjusted HR (95% CI)	Model 1, HR (95% CI)	Model 2, HR (95% CI)
**No asthma hospitalization before 15 years**	12491/26.26 × 10^6^	1 [reference]	1 [reference]	1 [reference]	9776/26.26 × 10^6^	1 [reference]	1 [reference]	1 [reference]
**Asthma hospitalization, 0–5 years**	147/24.15 × 10^4^	1.57 (1.34–1.85)	1.22 (1.04–1.43)	1.14 (0.96–1.34)	111/24.15 × 10^4^	1.39 (1.15–1.67)	1.13 (0.93–1.36)	1.08 (0.89–1.31)
**Asthma hospitalization 6–10 years**	37/63.40 × 10^3^	1.21 (0.87–1.66)	1.37 (0.99–1.89)	1.31 (0.94–1.82)	28/63.40 × 10^3^	1.17 (0.80–1.69)	1.12 (0.77–1.61)	1.11 (0.77–1.62)
**Asthma hospitalization 11–15 years**	30/38.97 × 10^3^	1.56 (1.09–2.23)	1.77 (1.24–2.54)	1.73 (1.21–2.47)	25/38.97 × 10^3^	1.69 (1.14–2.51)	1.72 (1.16–2.55)	1.62 (1.08–2.42)
**Asthma hospitalization <15 years**	214/34.26 × 10^4^	1.49 (1.30–1.71)	1.31 (1.14–1.49)	1.64 (1.43–1.89)	164/34.26 × 10^4^	1.38 (1.18–1.61)	1.19 (1.02–1.39)	1.14 (0.98–1.34)

*Note*: *Model 1:* adjusted for age, sex, calendar year. *Model 2:* adjusted for age, sex, calendar year, socioeconomic status, urban born, mother Swedish born, premature birth, birth order, maternal hospitalization due to infection during pregnancy, hospitalization due to respiratory infection before age 6, parental serious mental illness, maternal age, paternal age, maternal asthma history, paternal asthma history. PYAR, person years at risk; HR, hazard ratio; CI, confidence interval.

### Parental Asthma

Children of mothers with a history of hospitalization for asthma before their birth had increased rates of bipolar disorder (HR = 2.22, 95% CI = 1.76–2.80) (table 3). This association was maintained in the fully adjusted model (Model 3) (HR = 1.60, 95% CI = 1.27–2.02). There was also an association between maternal asthma admissions during pregnancy and bipolar disorder (fully adjusted HR = 1.73, 95% CI = 1.07–2.80) and between any report of asthma in the mother’s antenatal record and bipolar disorder (fully adjusted HR = 1.57, 95% CI = 1.32–1.88). Similarly, paternal pre-birth asthma admissions were associated with bipolar disorder in the offspring (fully adjusted HR = 1.44, 95% CI = 1.08–1.93).

**Table 3.** Bipolar Disorder and Schizophrenia Spectrum Disorders in Individuals Exposed to Parental Asthma

	Bipolar Disorder				Schizophrenia Spectrum Disorder			
	Cases Diagnosed/ PYAR	Unadjusted HR (95% CI)	Model 1, HR (95% CI)	Model 3, HR (95% CI)	Cases Diagnosed/ PYAR	Unadjusted HR (95% CI)	Model 1, HR (95% CI)	Model 3, HR (95% CI)
No maternal asthma hospitalization prebirth	12632/26.53 × 10^6^	1 [reference]	1 [reference]	1 [reference]	9899/26.53 × 10^6^	1 [reference]	1 [reference]	1 [reference]
Maternal asthma hospitalization prebirth	73/81.01 × 10^3^	2.22 (1.76–2.80)	1.65 (1.31–2.08)	1.60 (1.27–2.02)	41/81.01 × 10^3^	1.48 (1.08–2.02)	1.32 (0.96–1.80)	1.32 (0.97–1.80)
No maternal asthma hospitalization during pregnancy	12688/26.59 × 10^6^	1 [reference]	1 [reference]	1 [reference]	9931/26.59 × 10^6^	1 [reference]	1 [reference]	1 [reference]
Maternal asthma hospitalization during pregnancy	17/16.85 × 10^3^	2.32 (1.44–3.74)	1.90 (1.18–3.06)	1.73 (1.07–2.80)	9/16.85 × 10^3^	1.49 (0.77–2.86)	1.39 (0.72–2.67)	1.29 (0.65–2.41)
No maternal asthma prebirth (inc. antenatal record)	12575/26.46 × 10^6^	1 [reference]	1 [reference]	1 [reference]	9877/26.46 × 10^6^	1 [reference]	1 [reference]	1 [reference]
Maternal asthma prebirth (inc. antenatal record)	130/14.36 × 10^4^	2.69 (2.26–3.20)	1.61 (1.35–1.92)	1.57 (1.32–1.88)	63/14.36 × 10^4^	1.52 (1.18–1.96)	1.26 (0.98–1.62)	1.26 (0.97–1.62)
No paternal asthma hospitalization prebirth	12659/26.55 × 10^6^	1 [reference]	1 [reference]	1 [reference]	9925/26.55 × 10^6^	1 [reference]	1 [reference]	1 [reference]
Paternal asthma hospitalization prebirth	46/56.77 × 10^3^	2.01 (1.50–2.68)	1.48 (1.11–1.98)	1.44 (1.08–1.93)	15/56.77 × 10^3^	0.78 (0.45–1.33)	0.69 (0.40–1.17)	0.64 (0.38–1.08)

*Note*: *Model 1:* adjusted for age, sex, calendar year. *Model 3:* adjusted for age, sex, calendar year, socioeconomic status, urban born, mother Swedish born, birth order, mothers hospitalization due to infection during pregnancy, parental SMI, maternal age, paternal age, and other parent’s asthma status before birth. PYAR, person years at risk; HR, hazard ratio; CI, confidence interval.

In the unadjusted analysis, there was an association between maternal hospitalization for asthma and schizophrenia spectrum disorder (HR = 1.48, 95% CI = 1.08–2.02) and any maternal antenatal record of asthma and psychosis (HR = 1.52, 95% CI = 1.18–1.96). However, this association was attenuated after adjustment. There was no association between paternal asthma and schizophrenia spectrum disorder.

Limiting the cohort to children born after 1982 and additionally adjusting for maternal smoking did not dramatically alter the results for bipolar disorder or schizophrenia spectrum disorder (supplementary table 3).

## Discussion

To our knowledge, this is the first study to demonstrate an association between maternal asthma and offspring bipolar disorder. This association persists despite adjusting for a wide range of demographic and medical confounding factors. Additionally, we found an association between paternal asthma and bipolar disorder. We found less evidence for an association between maternal asthma pre-birth and schizophrenia spectrum disorder, and no evidence for an association with paternal asthma. This study also supports the previous finding that adolescent (11–15 years old) asthma is associated with both bipolar disorder and schizophrenia spectrum disorder. We found no association between early childhood asthma and bipolar disorder or schizophrenia spectrum disorder after accounting for important confounders. Taken as a whole, our results do not support the hypothesis that fetal and early childhood exposure to asthma are associated with developing SMI through inflammatory impacts on neurodevelopment or via other materno-fetal pathways. However, there may potentially be shared genetic architecture for asthma and SMI.

The riskiest period for childhood asthma appears to be adolescence. This finding is consistent across previous literature and our discordant sibling analysis. Although sibling analyses attempts to reduce unmeasured genetic or environmental confounding it does not rule it out and alternative, noncausal explanations remain. For example, the stress of physical illness and admission to hospital may result in increased SMI.^40^

The association between maternal asthma and bipolar disorder was consistent across a number of definitions of the exposure (ever hospitalized before birth, hospitalized during pregnancy and any self-report of asthma in the antenatal notes—which would include mothers with less severe asthma). However, the association between paternal asthma and bipolar disorder was unexpected and suggests that the increase in rates of bipolar disorder in the offspring of mothers with asthma is not materno-fetal in origin. Instead, this association may be genetic, epigenetic or result from unmeasured environmental factors. To the best of our knowledge, no study to date has investigated the shared heritability between asthma and bipolar disorder. Negative genetic correlations between forced expiratory volume (a polygenetic trait in the general population that is reduced in patients with asthma^41^) and cognitive abilities,^42,43^ as well as negative genetic correlations between bipolar disorder and cognition,^42,44^ suggest that there may exist a shared genetic basis for asthma and bipolar disorder. It is also unclear if the association is specific to bipolar disorder. An even stronger negative genetic correlation has been reported between cognitive abilities and schizophrenia,^42^ but may not include genes involved in asthma. Along with the study by Pedersen et al,^4^ our current observations indicate that shared genes may not fully explain the association between asthma during early adolescence and the later development of schizophrenia spectrum disorders.

We used national registers to study the entire population of Sweden, and the systematic routine collection of this data avoids recall bias. Given the size of the cohort, it is unlikely that we failed to find an association between admissions for early childhood asthma and bipolar disorder and schizophrenia spectrum disorder because of lack of power. However, we could have been underpowered for the parental exposure groups and supplementary analyses.

We studied severe asthma necessitating inpatient admission, and considering the heterogeneity of both the asthma phenotype and underlying inflammatory cell type and signaling abnormalities, our findings might not be generalizable to the entire spectrum of asthma patients. However, with regards to maternal asthma and bipolar disorder, we found a similar hazard ratio when we used a less severe definition of asthma (a history of asthma at antenatal checks). Asthma admission has been shown to have a positive predictive value of 93% in Swedish registers.^45^ In our study, approximately 2% of the cohort had an admission for asthma before the age of 15. Over the same period the prevalence of asthma increased in Sweden to 6%, but the proportion of “severe asthma” remained constant at 20% of cases.^46^ Previous studies have shown hospitalization in schizophrenia accurately reflects its true population prevalence, with 90% of those with the diagnosis being admitted at some point during their illness.^47^ Although less evidence is available on how accurately service-use in bipolar disorder and psychosis not diagnosed as schizophrenia reflects its true population prevalence, we surveyed both inpatient and outpatient records to maximize capture of this outcome. Missing data were not a threat to validity in analyses using the full cohort as only 1.3% were missing at least one data point. In the cohort born after 1982, 8.6% were missing maternal smoking information, but missingness was not associated with either SMI outcome. In both cases, analysis following listwise deletion should therefore be unbiased.^48^

We adjusted for a number of important confounders; however, there may be potential unmeasured and residual confounding in our fully adjusted models. For example, while we have attempted to adjust for it in this study, there is a strong association between childhood asthma and early childhood respiratory infections; this may have led to some exposure misclassification and residual confounding. Also, we were unable to account for corticosteroid use as this information was not available from Swedish population registers for the period of interest. Corticosteroid use could potentially be on the pathway between asthma and bipolar or schizophrenia spectrum disorders. We would hypothesize that all individuals hospitalized because of asthma would be exposed to corticosteroids of some kind. There is conflicting evidence about the effects of steroids in SMI. Although some studies suggest they acutely increase the risk of manic symptoms and psychosis,^49,50^ there are studies that find reduced longer-term psychosis and bipolar disorder risk in individuals exposed to corticosteroids.^6,51^ As such, we would only expect to see an association between corticosteroid use and psychosis if the medication use continued throughout the risk period for developing bipolar disorder or schizophrenia spectrum disorders. Beyond the asthma diagnosis, records of serum levels of inflammatory cytokines prenatally, during pregnancy, and from the neonate at delivery, could clarify our findings. Some of this data is potentially available from Swedish stored maternal sera or newborn blood spots^18^ but was not available for this study. Despite the advantages of our sibling analysis in terms of controlling for shared environmental and genetic confounders, there is potential for bias in this analysis from nonshared confounders and measurement error.^52^ In addition, sibling analyses will be less representative than analyses of the general population because inclusion requires participants to have one or more sibling and be discordant.^52^

This study strengthens the evidence that severe asthma during adolescence increases the risk of SMI. It suggests that there is a stronger association between parental asthma and bipolar disorder compared to schizophrenia spectrum disorders. This requires further investigation, but if replicated has implications for our understanding of the differential mechanisms which are at play in the etiology of different SMI. Shared genetic factors potentially link asthma and SMI.

## Supplementary Material

Supplemental Table
