# Supplementary material for: Childhood and Parental Asthma, Future Risk of Bipolar Disorder and Schizophrenia Spectrum Disorders: A Population-Based Cohort Study
Source: Schizophr Bull. 2018 Mar 9;45(2):360–8. doi: 10.1093/schbul/sby023 (PMC6403048; doi:10.1093/schbul/sby023)
Supplement: Supplemental Table [file sby023_suppl_supplemental_table.doc]

## Supplementary Tables

### Supplementary Table 1. ICD codes

| **DIAGNOSIS** | **ICD10 CODES** | **ICD9 CODES** | **ICD8 CODES** |
| --- | --- | --- | --- |
| **Asthma** | J45.0; J45.0A; J45.0B; J45.0W; J45.1; J45.1A; J45.1W; J45.8; J45.9; J46 | 493A; 493B; 493X | 493,00; 493,02; 493,08; 493,09 |
| **Bipolar disorder** | F30.0; F30.1; F30.2; F30.8; F30.9; F31.0; F31.1; F31.2; F31.3; F31.4; F31.5; F31.6; F31.7; F31.8; F31.9 | 296A; 296C; 296D; 296E; 296W; 296X | 296,10; 296,20; 296,30; 296,88; 296,99 |
| **Schizophrenia spectrum disorder** | F20.0; F20.1; F20.2; F20.3; F20.4; F20.5; F20.6; F20.8; F20.9; F21; F22.0; F22.8; F22.9; F23.0; F23.1; F23.2; F23.3; F23.8; F23.9; F24; F28; F29 | 295A; 295B; 295C; 295D; 295E; 295G; 295W; 295X; 297B; 297C; 297D; 297W; 297X; 298B; 298C; 298E; 298W; 298X | 295,00; 295,10; 295,20; 295,30; 295,40; 295,60; 295,80; 295,99; 297,00; 297,10; 297,98; 298,10; 298,20; 298,30; 298,99; 299,99 |
| **Severe mental illness, (including bipolar disorder, schizophrenia, schizoaffective disorder and non-affective non-organic psychoses)** | F30.0; F30.1; F30.2; F30.8; F30.9; F31.0; F31.1; F31.2; F31.3; F31.4; F31.5; F31.6; F31.7; F31.8; F31.9; F20.0; F20.1; F20.2; F20.3; F20.4; F20.5; F20.6; F20.8; F20.9; F25.0; F25.1; F25.2; F25.8; F25.9; F21.9; F22.0; F22.8; F22.9; F23.0; F23.1; F23.2; F23.3; F23.8; F23.9; F24.9; F28.9; F29.9 | 296A; 296B; 296C; 296D; 296E; 296W; 296X; 295A; 295B; 295C; 295D; 295E; 295F; 295G; 295H; 295W, 295X; 298A; 297B; 297C; 297D; 297W; 297X; 298B; 298C; 298E; 298W; 298X | 296,00; 296,10; 296,20; 296,30; 296,88; 296,99; 295,00; 295,10; 295,20; 295,30; 295,40; 295,50; 295,60; 295,70; 295,80; 295,99; 297,00; 297,10; 297,98; 298,00; 298,10; 298,20; 298,30; 298,99; 299,99 |
| **Upper or lower acute or chronic respiratory tract infection** | J009; J010; J011; J012; J013; J014; J018; J019; J020; J028; J029; J030; J038; J039; J040; J041; J042; J050; J051; J060; J068; J069; J099; J100; J101; J108; J110; J111; J118; J120; J121; J122; J123; J128; J129; J139; J149; J150; J151; J152; J153; J154; J155; J156; J157; J158; J159; J160; J168; J170; J171; J172; J173; J178; J180; J181; J182; J188; J189; J200; J201; J202; J203; J204; J205; J206; J207; J208; J209; J210; J211; J218; J219; J229; J310; J311; J312; J320; J321; J322; J323; J324; J328; J329; J350; J351; J352; J353; J358; J359; J369; J370; J370A; J370B; J370C; J370W; J370X; J371; J409; J410; J411; J418; J429; J00; J01; J02; J03; J04; J05; J06; J09; J10; J11; J12; J13; J14; J15; J16; J17; J18; J20; J21; J22; J31; J32; J35; J36; J37; J40; J41; J42 | 460X; 461A; 461B; 461C; 461D; 461W; 461X; 462X; 463X; 464A; 464B; 464C; 464D; 464E; 465A; 465W; 465X; 466A; 466B; 472A; 472B; 472C; 473A; 473B; 473C; 473D; 473W; 473X; 474A; 474B; 474C; 474W; 474X; 475X; 476A; 476B; 480A; 480B; 480C; 480W; 480X; 481X; 482A; 482B; 482C; 482D; 482E; 482J; 482W; 482X; 483X; 484A; 484B; 484C; 484D; 484E; 484F; 484G; 484H; 484W; 485X; 486X; 487A; 487B; 487W; 490X; 491A; 491B; 491C; 491W; 491X; 460; 461; 462; 463; 464; 465; 466; 472; 473; 474; 475; 476; 480; 481; 482; 483; 484; 485; 486; 487; 490; 491 | 460,99; 461,00; 461,01; 461,02; 461,03; 461,04; 461,09; 462,01; 462,02; 462,09; 463,01; 463,09; 464,01; 464,02; 464,03; 464,09; 465,99; 466,99; 470,99; 471,01; 471,09; 472,01; 472,02; 472,09; 473,99; 474,99; 480,99; 481,99; 482,01; 482,10; 482,20; 482,30; 482,98; 483,99; 484,99; 485,01; 485,02; 485,09; 486,01; 486,09; 490,99; 491,01; 491,02; 491,04; 491,09; 501,99; 502,00; 502,10; 503,00; 503,01; 503,02; 503,03; 503,04; 503,05; 503,06; 503,08; 503,09; 506,01; 506,09; 508,00; 508,01; 508,02; 508,03 |

### Supplementary Table 2. Discordant siblings analysis

|  | **Bipolar disorder** |  |  |  | **Schizophrenia spectrum disorder** | | | |  | | |
| --- | --- | --- | --- | --- | --- | --- | --- | --- | --- | --- | --- |
|  | **Cases diagnosed/PYAR** | **Unadjusted HR (95% CI)** | **Model 1**  **HR (95% CI)** | **Model 2**  **HR (95% CI)** | **Cases diagnosed/PYAR** | **Unadjusted HR (95% CI)** | **Model 1**  **HR (95% CI)** | **Model 2**  **HR (95% CI)** |  |  |  |
| **No asthma hospitalisation before 15y (N=39462)** | 241/3.85x10^5^ | 1  [reference] | 1  [reference] | 1  [reference] | 161/3.85x10^5^ | 1  [reference] | 1  [reference] | 1  [reference] |  |  |  |
| **Asthma hospitalisation**  **0-5y (N=22229)** | 103/1.69x10^5^ | 1.21  (0.90-1.63) | 1.15  (0.82-1.61) | 1.18  (0.81-1.73) | 72/1.69x10^5^ | 1.04  (0.74-1.47) | 0.93  (0.65-1.34) | 0.71  (0.46-1.07) |  |  |  |
| **Asthma hospitalisation**  **6-10y (N=3308)** | 29/4.18x10^4^ | 0.90  (0.52-1.56) | 0.96  (0.51-1.81) | 1.98  (0.51-1.87) | 17/4.18x10^4^ | 1.26  (0.62-2.55) | 1.25  (0.60-2.59) | 1.35  (0.63-2.86) |  |  |  |
| **Asthma hospitalisation 11-15y (N=1948)** | 19/2.46x10^4^ | 1.37  (0.68-2.76) | 1.61  (0.71-3.62) | 1.61  (0.72-3.63) | 16/2.46x10^4^ | 2.82  (1.09-7.29) | 3.11  (1.19-8.16) | 2.87  (1.05-7.79) |  |  |  |
| **Asthma hospitalisation**  **<15y (N=27485)** | 151/2.35x10^5^ | 1.16  (0.90-1.48) | 1.15  (0.87-1.53) | 1.18  (0.87-1.62) | 105/2.35x10^5^ | 1.19  (0.88-1.60) | 1.11  (0.82-1.51) | 0.95  (0.68-1.34) |  |  |  |

PYAR: person years at risk, HR: hazard ratio, CI: confidence interval

**Model 1:** adjusted for age, sex, calendar year

**Model 2:** adjusted for age, sex, calendar year, urban born, premature birth, birth order, maternal hospitalisation due to infection during pregnancy, hospitalisation due to respiratory infection before aged 5, maternal age, paternal age

### Supplementary Table 3. Fully adjusted hazard ratios for cohort born after start of 1982 (complete smoking records)

|  | **Adjusted^1^ HR (95%CI)** | |  | | |
| --- | --- | --- | --- | --- | --- |
|  | **Bipolar disorder** | **Schizophrenia spectrum** |  |  |  |
| **No asthma hospitalisation before 15y (N=1384764)** | 1 [reference] | 1 [reference] |  |  |  |
| **Asthma hospitalisation**  **0-5y (N=28966)** | 1.14 (0.94-1.39) | 1.04 (0.82-1.31) |  |  |  |
| **Asthma hospitalisation**  **6-10y (N=2704)** | 0.80 (0.41-1.53) | 0.82 (0.39-1.72) |  |  |  |
| **Asthma hospitalisation 11-15y (N=1556)** | 1.73 (0.96-3.13) | 1.14 (0.47-2.74) |  |  |  |
| **Asthma hospitalisation**  **<15y (N=33226)** | 1.14 (0.96-1.36) | 1.02 (0.82-1.27) |  |  |  |
| **No maternal asthma hospitalisation pre-birth (N=1409509)** | 1 [reference] | 1 [reference] |  |  |  |
| **Maternal asthma hospitalisation pre-birth (N=8,481)** | 1.50 (1.13-1.99) | 1.39 (0.95-2.02) |  |  |  |
| **No maternal asthma hospitalisation during pregnancy (N=1416605)** | 1 [reference] | 1 [reference] |  |  |  |
| **Maternal asthma hospitalisation during pregnancy (N=1385)** | 2.16 (1.25-3.72) | 0.76 (0.25-2.37) |  |  |  |
| **No maternal asthma pre-birth (inc. antenatal record) (N=1392434)** | 1 [reference] | 1 [reference] |  |  |  |
| **Maternal asthma pre-birth (inc. antenatal record) (N=25556)** | 1.49 (1.22-1.83) | 1.33 (0.99-1.78) |  |  |  |
| **No paternal asthma hospitalisation pre-birth (N=1411752)** | 1 [reference] | 1 [reference] |  |  |  |
| **Paternal asthma hospitalisation pre-birth (N=6238)** | 1.50 (1.07-2.10) | 0.78 (0.43-1.42) |  |  |  |

1. Childhood asthma exposure: adjusted for age, sex, calendar year, SES, urban born, mother Swedish born, premature birth, birth order, maternal hospitalisation due to infection during pregnancy, hospitalisation due to respiratory infection before aged 6, parental SMI, maternal age, paternal age, maternal asthma history, paternal asthma history, maternal smoking

Parental asthma exposure adjusted for age, sex, calendar year, SES, urban born, mother Swedish born, birth order, mothers hospitalisation due to infection during pregnancy, parental SMI, maternal age, paternal age, and other parent’s asthma status before birth, maternal smoking
